# Epilepsy management in pregnant HIV+ women in sub-Saharan Africa, clinical aspects to consider: a scoping review

**DOI:** 10.1186/s12916-020-01799-0

**Published:** 2020-11-17

**Authors:** Sonia Menon, Lenka Benova, Hillary Mabeya

**Affiliations:** 1 Instiute of Tropical Medicine Antwerp, Antwerp, Belgium; 2 Moi University/Gynocare Fistula Centre, El Doret, Kenya

**Keywords:** HIV, Epilepsy, Antiretroviral treatment, Antiepileptics, Pregnancy, Women of reproductive age

## Abstract

**Background:**

Since the introduction of highly active antiretroviral therapy (HAART), acquired immune deficiency syndrome (AIDS) related mortality has markedly declined. As HAART is becoming increasingly available, the infection with human immunodeficiency virus (HIV+) in sub-Saharan Africa (SSA) is becoming a chronic condition. While pregnancy in HIV+ women in SSA has always been considered a challenging event for the mother and the fetus, for pregnant HIV+ women also diagnosed with epilepsy (WWE), there are additional risks as HIV increases the odds of developing seizures due to the vulnerability of the central nervous system to other infections, immune dysfunction, and overall metabolic disturbances. In light of a growing proportion of HIV+ WWE on HAART and an increasing number of pregnant women accessing mother-to-child transmission of HIV programs through provision of HAART in SSA, there is a need to develop contextualized and evidenced-based clinical strategies for the management of epilepsy in this population. In this study, we conduct a literature scoping review to identify issues that warrant consideration for clinical management.

**Result:**

Twenty-three articles were retained after screening, which covered six overarching clinical aspects: status epilepticus (SE), Stevens-Johnson syndrome/toxic epidermal necrolysis (SJS/TEN), dyslipidemia, congenital malformation (CM), chronic kidney disease (CKD), and neurological development. No studies for our population of interest were identified, highlighting the need for a cautionary approach to be employed when extrapolating findings.

**Conclusion:**

High risks of CM and drug interactions with first-line antiepileptic drugs (AEDs) warrant measures to increase the accessibility and choices of safer second-line AEDs. To ensure evidence-based management of epilepsy within this population, the potential high prevalence of SE, CKD, dyslipidemia, and SJS/TEN and the cumulative effect of drug-drug interactions should be considered. Further understanding of the intersections between pregnancy and drug-drug interactions in SSA is needed to ensure evidenced-based management of epilepsy in pregnant HIV+ WWE. To prevent SE, the barriers for AED treatment adherence in pregnant HIV+ women should be explored. Our review underscores the need to conduct cohort studies of HIV+ WWE in reproductive age over time and across pregnancies to capture the cumulative effect of HAART and AED to inform clinical management.

## Background

According to the World Health Organization (WHO), there were approximately 38 million people across the globe with human immunodeficiency virus (HIV) or acquired immune deficiency syndrome (AIDS) at the end of 2019 [1]. The burden disproportionately affects sub-Saharan Africa (SSA) where over two thirds of all people living with HIV reside [1] and where women and girls account for 59% of all new HIV infections [2]. Since the introduction of highly active antiretroviral therapy (HAART), HIV is becoming a chronic condition for the 16.4 million people on the drug regimen in SSA [3]. Furthermore, around 1.4 million HIV infections among children were prevented between 2010 and 2018 due to the implementation of services for the prevention of mother-to-child transmission (PMTCT) of HIV [4].

Use of HAART has entailed an excess risk of seemingly unrelated non-AIDS conditions, such as hyperlipidemia [5], Stevens-Johnson syndrome/toxic epidermal necrolysis (SJS/TEN) [6], chronic kidney disease (CKD) [7], and cardiovascular diseases [8, 9]. Furthermore, HIV+ individuals are at heightened risk for developing seizures due to the vulnerability of the central nervous system to opportunistic diseases, immune dysfunction, and metabolic disturbances [10]. In addition, some drugs indicated for the treatment of HIV or HIV-associated infections and coexisting metabolic derangements may also induce seizures [10]. A recent meta-analysis reported a high pooled prevalence of seizures in a general HIV+ population of 62 per 1000 (95% confidential interval [CI] 37.3–93.1) [11], in contrast to a pooled lifetime prevalence of 7.60 per 1000 persons (95% CI 6.17–9.38) reported in a general population [12].

Epilepsy is diagnosed after a person experiences two unprovoked seizures more than 24 h apart [13]. The majority of seizures in HIV+ individuals are generalized seizures [14] involving both cerebral hemispheres, which may need emergency medical attention. With an increasing burden of chronic HIV infections in low-income countries (LIC), HIV infection has become an important risk factor of epilepsy and status epilepticus (SE) [15].

Nearly 80% of people with epilepsy live in LIC, where it is estimated that in an ideal scenario up to 70% of people living with epilepsy are seizure-free if properly diagnosed and treated [16].

In 2019, in HIC, there were estimated to be 49 per 100, 000 people diagnosed with epilepsy each year, whereas in low- to middle-income countries, estimates were at 139 per 100, 000 [17]. It is noteworthy that the epilepsy treatment gap, defined as the proportion of people with epilepsy who require treatment but do not receive it [18], in the general population varies from 23% in Senegal [19] to 100% in Uganda, Tanzania, Gambia, and Togo [18].

In most LIC, the first-line enzyme-inducing antiepileptic drug (EI-AED), phenobarbital, is the only AED in widespread use [20]. Although other first-line AEDs, including phenytoin, carbamazepine, and valproic acid, are commonly available, they entail higher out-of-pocket costs [21]. The first-line EI-AEDs, metabolized predominantly by the liver, are problematic for HIV+ individuals as they are inducers of the cytochrome P450 hepatic enzyme system and thus interact with anti-infective drugs, including HAART [10].

In 2018, the WHO issued interim guidance for HAART recommending two nucleoside reverse transcriptase inhibitors (NRTIs) tenofovir, lamivudine, paired with dolutegravir to replace nevirapine (NRTI) as the preferred first-line regimen for HIV treatment, also in adolescent girls and women who are pregnant [22]. However, dolutegravir effectiveness has been shown to be reduced in patients treated with phenobarbital [23], and there have also been reports of interactions with valproic acid [24]. This gave rise to the recommendation that to prevent virologic failure, concomitant use of HAART regimens that include protease inhibitors (PI) or NRTIs and EI-AED should be avoided [25].

Compared to these EI-AEDs, second-line broad-spectrum non-EI-AEDs including levetiracetam and lamotrigine have fewer drug interactions [26]. However, these second-line AEDs for generalized and partial seizures are not widely available in SSA [20]. Currently, the only second-line AED for partial and generalized seizures for pregnant women in the WHO Essential List of Medicines is lamotrigine [27], which undergoes extensive metabolism in the liver [28].

Apart from drug interactions with HAART and anti-infective agents, first-line EI-AEDs are significantly more teratogenic than second-line AEDs [29]. The WHO recommends carbamazepine for pregnant women with epilepsy (WWE) if no second-line AED is available [17], due to its slightly lower prevalence of congenital malformation (CM) (5.5%), compared to phenobarbital (6.5%), and lamotrigine and levetiracetam (2.9% and 2.8%, respectively) [30] (Table 1).

**Table 1:** Table with safety profile of AED in pregnant women

AED	Indication	Neurological development after in utero exposure	% of fetal malformation	Enzyme inducing	SJS/TEN	Dyslipidemia
Phenobarbital	Broad spectrum AED	No statistically significant association with adverse neurological outcomes and autism^1^	6.50%^2^	Yes	Yes	Yes
Phenytoin	Broad spectrum AED	No statistically significant association with adverse neurological outcomes and autism ^1^	6.4%^2^	Yes	Yes	Yes
Carbamazepine	Broad spectrum AED	No statistically significant association with adverse neurological outcomes and autism ^1^	5.50% ^2^	Yes	Yes	Yes
Valproic acid	Broad spectrum AED	Pooled OR of adverse neurodevelopmental outcomes and autism compared to women who did not receive AED during pregnancy (OR=7.40; CI :95% 3.00 to 18.46); (OR:17.29; 95%CI: 2.40-217.60) respectively ^1^	10.30%^2^	No	Yes	No evidence
**Second line AED**			Absence of AED exposure: 2.6% ^2^			
Lamotrigine	Broad spectrum AED	Pooled OR of autism compared to women who did not receive AED during pregnancy (OR: 8.88; 95%CI: 1.28-112.00)^1^	2.90% ^2^	No	Yes	No evidence
Levetiracetam	Broad spectrum AED	No statistically significant association with adverse neurological outcomes and autism^1^	2.80% ^2^	No Not enzyme inducing, not associated with clinically relevant drug interactions	No evidence	No evidence

^1^Veroniki, et al. [31]. Published 2017 Jul 20. doi:10.1136/bmjopen-2017-017248

^2^Tomson et al. [30]

When treating WWE, clinicians face the additional dilemma that valproic acid, a first-line AED recommended for HIV+ individuals in LIC as it is not enzyme inducing [10], is associated with the highest prevalence of CM (10.3%) [30]. In addition, given the geographic overlap with other endemic diseases, a non-negligible proportion of pregnant HIV+ WWE may concomitantly receive treatment with other potentially teratogenic drugs, including anti-TB drugs [32]. Therefore, epilepsy management during pregnancy, particularly in HIV+ women, requires an equilibrium between seizure control, viral load control, co-infection prevention, and risk minimization for the WWE and their fetuses [33].

With a large number of HIV+ women who will be on HAART prior to getting pregnant and HIV+ pregnant women embarking on PMTCT programs, there is a need to develop evidenced-based clinical guidelines on epilepsy management in pregnant HIV+ women. This review seeks to identify aspects to be considered in clinical management of epilepsy in pregnant HIV+ women in SSA. Specifically, it explores clinical aspects which intersect between HIV, HAART, AED, and pregnancy.

## Methods

The methods were organized according to the seven following stages using a methodological framework for scoping reviews [34].

### Search strategy

A scoping review was performed to characterize the current understanding about the management of epilepsy in pregnant HIV+ women SSA. Literature searches were performed on PubMed on August 4, 2020. January 1997 onwards was selected as a starting date to limit the publications to the combined HAART era. Different sets of keywords and Boolean terms were used to identify literature, e.g., dyslipidemia AND pregnan* AND antiepileptic drugs AND antiretroviral AND Africa; dyslipidemia AND antiretroviral therapy AND pregnan* AND Africa; dyslipidemia AND antiretroviral therapy AND Africa; dyslipidemia AND HIV AND Africa (Additional file 1: Annex 1). Reference lists from retrieved publications were reviewed to identify additional manuscripts not captured by the search.

Literature reporting on the pooled burden pertaining to all six clinical aspects explored in this review was searched for our population of interest, along with risk factors. If no studies were identified for this specific population, a stepwise search process was undertaken, omitting one key term in each subsequent search. An additional hand-search was performed on the reference list of the retrieved literature.

### Inclusion criteria

Inclusion criteria are as follows: observational prospective, retrospective, and cross-sectional studies; quasi- and randomized controlled trials (RCTs); pre- and post-studies without a control group; systematic reviews; and/or meta-analyses reflecting the most current pooled evidence.

### Exclusion criteria

Exclusion criteria are as follows: case studies; case series; commentaries; studies included within the most current pooled evidence; studies focusing on children, older adults, and HAART-naïve patients; studies whose primary endpoints are not the ones of interest; and non-English literature.

### Research question

This scoping review aims to answer the following question: What are the clinical aspects which should be considered to provide safe and effective clinical management of pregnant HIV+ WWE on HAART in SSA?

### Study selection

We used the PICO model to answer our research question.

#### Population

Pregnant HIV+ WWE in SSA.

#### Intervention

The use of HAART and AEDs in pregnant HIV+ WWE.

#### Comparison

HIV+ WWE on AED, with or without a control group.

#### Outcome(s)

The outcomes are safety of the concomitant use of AED/HAART use in pregnant women, and adequate management of AED in pregnant HIV+ WWE on HAART.

### Extraction of data

Microsoft Excel 2016 was used to gather information on author(s), year of publication, study location and population, study design, main exposure of interest and outcome, and key findings.

### Synthesis of findings

Key findings were broken down into specific categories, derived from the articles rather than a predefined framework. We adhered to the Preferred Reporting Items for Systematic Reviews and Meta-analyses—Extension for Scoping Reviews (PRISMA-ScR) checklist and guidelines (Additional file 2: Annex 2).

## Results

**Fig. 1:**
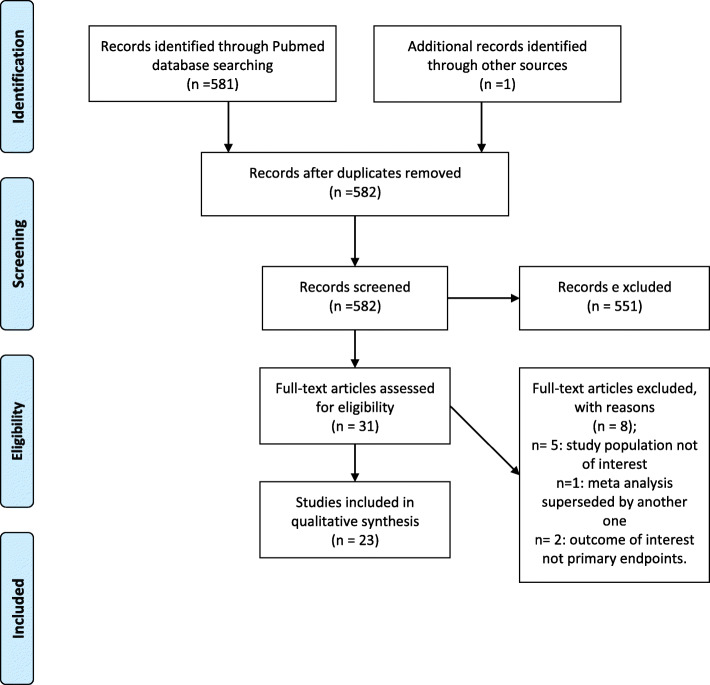
PRISMA flow diagram

Using the broadest search terms, of the 581 articles retrieved, 23 studies were retained; these included 19 original research articles and 4 systematic reviews and/or meta-analyses (Fig. 1). No articles on pregnant HIV+ WWE on HAART and AEDs in SSA were retrieved; only one study on HIV+ individuals on HAART and AEDs in SSA was identified. We identified six overarching clinical aspects: SE, STJ/TEN, dyslipidemia, CM, CKD, and neurological development, which intersect with pregnancy, AED treatment, and HAART (Table 2).

**Table 2:** Summary of studies included in the review by outcome of interest

First author (year)	Country or geographical area	Study design and sample size	Population and exposure(s) of interest	Main outcome(s) of interest	Main results and remarks
Elafros (2017) [35]	Zambia	Prospective observational cohort study (*N* = 145)	Patients on both HAART and AED	Adherence at 6 months	Suboptimal adherence at 6 months with 18 out of 33 (56%) participants on HAART experiencing more than 1 week lapse in AED supply. At 2 weeks, a greater proportion of participants on HAART and EI-AED participants reported increased nausea or vomiting compared with baseline (*p* < 0.05).
Knight (2015) [36]	South Africa	Prospective cohort study (*N* = 22)	HIV+ pregnant women with SJS/TEN	Maternal outcome for both the mother and fetus	Nevirapine was the offending drug in 21/22 (95%) cases. TEN was associated with poorer fetal outcomes. SJS/TEN-associated mortality is not increased in HIV+ pregnant women. Maternal SJS/TEN does not seem to commonly manifest in the fetus.
Saka (2013) [37]	Benin, Burkina Faso, Central African Republic, and Togo	Retrospective cross-sectional multicentric study (*N* = 177)	Patients with SJS/TEN of which 69% were HIV	SJS/TEN	Sex ratio (M/F) was 0.6, and the drugs responsible for SJS/TEN were dominated by antibacterial sulfonamides (38.4%) and nevirapine (19.8%), followed by tuberculosis drugs (5.6%).
Irungu (2017) [38]	Kenya	Cross-sectional study (*N* = 115)	Patients with severe cutaneous reactions	SJS/TEN	Females represented 59.1% of patients. The most common drugs implicated were sulfonamides (26.1%) and nevirapine (15.7%).
Kannenberg (2012) [39]	South Africa	Prospective observational cohort study (*N* = 75)	Patients admitted with TEN/SJS	SJS/TEN	Most patients with TEN/SJS were HIV+ (*n* = 59) and female (*n* = 51). Cotrimoxazole (24%) and nevirapine (29.3%) were the most common precipitants of TEN/SJS.
Dube (2013) [40]	South Africa	Retrospective study (matched case control study) (*N* = 36 of which 6 cases and 30 controls)	HIV+ pregnant women taking nevirapine-based regimens	SJS	Pregnancy increased the chances of developing SJS 14-fold (OR 14.28; 95% CI 1.54–131.82).
Marazzi (2006) [41]	SSA	Retrospective observational cohort study (*N* = 703)	HIV+ pregnant women treated with nevirapine-based regimens	SJS	Nevirapine-containing regimens in pregnant woman appear safe with only 1.1% developing SJS.
Noubiap (2018) [42]	SSA	Meta-analysis of 177 studies (*N* = 294,063)	General population and HIV+ on HAART	Dyslipidemia	Prevalence of dyslipidemia based on total cholesterol concentrations (25.47%; 95% CI 19.41–32.03%) (*N* = 6527), HDL cholesterol (45.6%; 95% CI 34.11–57.30%) (*N* = 5590), LDL cholesterol (24.30%; 95% CI 14.71–35.38%) (*N* = 5671), and triglycerides (19.68%; 95% CI 14.77–25.09%) (*N* = 6766).
Omo-Aghoja (2016) [43]	Nigeria	Matched case control nested within a cohort study (*N* = 154 pregnant women; *N* = 151 non-pregnant)	Pregnant HIV+ women	Dyslipidemia	Higher mean of very-low-density lipoprotein mg/dl was reported in pregnant women 67.79 (SD 162.75) versus non-pregnant women (*p* = 0.045).
Mehta (2019) [44]	South Africa	Prospective observational cohort study (*N* = 10,417 of which 4013 were HIV+)	Infants born to HIV+ women on nevirapine-based regimens in the first trimester	Congenital malformation	First trimester exposure to nevirapine was associated with an increased risk of CM (risk ratio 9.28; 95% CI 2.3–37.9) compared to births not exposed to ART in the first semester. CM rates in births exposed to efavirenz during the first trimester were similar to births not exposed to HAART (RR 0.87; 95% CI 0.12–6.4).
Liu (2014) [45]	South Africa/Zambia	Prospective observational cohort (*N* = 600)	Infants born to HIV+ women on HAART	Congenital malformation	Overall CM at delivery detected was 6.4% in South Africa and 5.6% in Zambia.
Raesima (2019) [46]	Botswana	Birth outcomes surveillance study (*N* = 3076)	Infants born to HIV+ women on dolutegravir-based regimens	Congenital malformation	A slightly higher prevalence of neural-tube defects among deliveries in HIV+ mothers who had been taking dolutegravir at the time of conception than among deliveries in which the mothers were HIV− (< 1%).
Zash (2019) [47]	Botswana	Birth outcomes surveillance study (*N* = 1683 deliveries)	Infants born to HIV+ women on dolutegravir-based regimens	Congenital malformation	Difference of prevalence of 0.20% (95% CI 0.01–0.59) in neural-tube defects among deliveries when dolutegravir is taken at conception compared to non-dolutegravir-based HAART. Also, there were major external structural defects observed in 0.95% of deliveries among women exposed to dolutegravir at conception versus 0.68% in women not exposed to dolutegravir.
Ekouevi (2011) [48]	West Africa	Retrospective cross-sectional study (*N* = 344)	Pregnant HIV+ women on efavirenz- or nevirapine-based regimens during the first trimester of pregnancy	Congenital malformation	No visible congenital malformation was observed in women exposed to efavirenz or nevirapine.
Gibb (2012) [49]	Uganda/Zimbabwe	RCT (*N* = 382 pregnancies)	Pregnant HIV+ women on tenofovir-based regimens	Congenital malformation	No increase in congenital, renal, or growth abnormalities was observed with in utero tenofovir exposure.
Ford (2010) [50]	Global/SSA	Meta-analysis with 12 studies (*N* = 9835)	Pregnant HIV+ women on efavirenz-based regimens during the first trimester of pregnancy	Congenital malformation	No differences in overall risks of CM between women on efavirenz versus non-efavirenz-containing regimens during the first trimester of pregnancy (relative risk 0.78, 95% CI 0.56–1.08).
Baynes (2019) [51]	SSA	Meta-analysis of 15 studies (*N* = 86,879)	HIV+ patients on HAART	Prevalence of CKD	Overall pooled prevalence was 6.42% (95% CI 5.2–7.6%), and the majority of patients with CKD were diagnosed with stage 3.
Mtisi (2019) [52]	SSA	Systematic review of 31 studies (*N* = 106,406)	HIV+ adults on tenofovir-based HAART	Decline of renal function	Some studies reported statistically significant renal function decline, but the clinical significance of this effect was not enough to contraindicate its continued use in HAART regimens.
Cassidy (2019) [53]	Botswana	Prospective observational cohort study (*N* = 493)	Pregnant HIV+ women on efavirenz-based regimens	Neurodevelopmental outcomes	Adjusted mean scores for the efavirenz-exposed group were poorer compared to the efavirenz-unexposed group on Bayley-III Receptive Language (21.5 versus 22.5, *p* = 0.05), Developmental Milestones Checklist Locomotor (30.7 versus 32.0, *p* < 0.01), Fine Motor scales (17.8 versus 19.2, *p* < 0.01), Profile of Social Emotional Development (11.7 versus 9.9, *p* = 0.02), and Developmental Milestones Checklist Language scale (17.6 versus 16.5, *p* = 0.01).
Chaudhury (2018) [54]	Botswana	Prospective study nested within one observational cohort and one interventional (*N* = 598)	HIV exposed and uninfected children exposed in utero to HAART versus zidovudine monotherapy	Neurodevelopmental outcomes	Neurodevelopmental outcomes at 24 months of age were similar in maternal HAART exposed versus zidovudine exposed HIV exposed uninfected children.
Chaudhury (2017) [55]	Botswana	Prospective observational cohort study (*N* = 670)	In utero HIV-exposed uninfected children, of which 36% were exposed in utero to maternal HAART and 64% to zidovudine	Neurodevelopmental outcomes	No evidence suggesting an adverse impact of in utero HIV and maternal HAART exposure on early age neurodevelopment.
Wedderburn (2019) [56]	South Africa	Prospective observational cohort study (*N* = 1225)	HIV-exposed uninfected children on HAART	Neurodevelopmental outcomes	Uninfected children exposed to maternal HIV infection and HAART have increased odds of receptive and expressive language delays at 2 years (aOR 1.96, 95% CI 1.09–3.52; aOR 2.14, 95% CI 1.11–4.15, respectively).
Kacanek (2018) [57]	Botswana	RCT (*N* = 197)	HIV-exposed uninfected children with in utero exposure to NRTI containing HAART	Neurodevelopmental outcomes	Neurodevelopmental outcomes in 24-month-old HIV-exposed uninfected children of HIV+ mothers with baseline CD4 ≥ 200 were similar in those randomized to a dual–NRTI + protease inhibitor-based compared to a triple-NRTI-based HAART regimen.

### Status epilepticus

There is no study examining AED adherence, in pregnant HIV+ women on both HAART and AED.

SE, HIV, and pregnancy intersect. SE in LIC has an infectious etiology or in those with already established epilepsy can be attributed to AED noncompliance [58]. There is evidence suggesting that HIV is a significant risk factor for developing SE, with a multicentric cross-sectional study exploring the association between convulsive SE among HIV+ individuals with active convulsive epilepsy in South Africa, Uganda, and Kenya reporting an adjusted odds ratio ([aOR] 2.1; 95% CI 1.04–4.31) [59].

Also, SE can pose as a significant risk to both mother and fetus [60].

Of the six articles retrieved on SE and HIV, there was no study assessing the burden of SE in a HIV+ population on HAART. We retrieved only one article on AED adherence in HIV+ adults on HAART. One cohort study in Zambia [35] reported a suboptimal adherence at 6 months, resulting in 18 out of 33 (56%) participants on HAART experiencing greater than 1-week lapse in AED supply, with participants reporting increased nausea or vomiting compared with baseline (*p* < 0.05).

### SJS/TEN

There were no studies examining the impact of concomitant use of HAART and AED on SJS/TEN in pregnant HIV+ women in SSA.

SJS/TEN, AED, HAART, and pregnancy interact. It is well established that HIV+ persons have a higher incidence of SJS/TEN [61] with evidence suggesting poorer fetal outcomes in HIV+ women with SJS/TEN [36]. Treatment with commonly used AEDs such as carbamazepine, phenytoin, phenobarbital, and lamotrigine is considered to increase the risk of SJS or TEN [62]. A meta-analysis reported an incidence of skin rash with lamotrigine of almost 10% of persons from prospective studies [63] from high-income countries (HIC). Similarly, a recent review suggested that HAART, being implicated in 90% of cases, constitutes the most common putative medications in pregnant women [64].

The prevalence of the allele, human leukocyte antigen B gene*∗15:02*, which is strongly associated with an increased risk of SJS and TEN, in patients taking carbamazepine [65, 66] and related drugs, including phenytoin and lamotrigine [67], is higher in people of African origin than in Caucasians (0.21% versus 0.001%, respectively) (www.allelefrequencies.net). Consequently, it is possible that the risk of SJS/TEN associated with these AEDs in SSA may be higher.

We retrieved 109 articles examining SJS/TEN in HIV+ individuals, of which five retrospective studies [37–41] and a prospective study were eligible [36]. No meta-analysis was retrieved on the burden of SJS/TEN in HIV+ individuals on HAART or without in SSA. Three retrospective studies found nevirapine to be among the most common precipitants [37–39].

Two retrospective studies [40, 41] and a prospective study [36] examined data in HIV+ pregnant women on HAART. A matched case control study in South Africa reported that pregnancy increased the chances of developing SJS 14-fold (OR 14.28; 95% CI 1.54–131.82) [40]. While a retrospective cohort study from a SSA reported that only 1.1% of the 703 HIV+ women on nevirapine developed SJS [41], in a prospective cohort study of pregnant HIV+ women in South Africa, nevirapine was found to be the offending drug in 95% of cases [36].

### Dyslipidemia

No studies were retrieved assessing dyslipidemia among pregnant HIV+ WWE on HAART and AEDs in SSA.

Dyslipidemia, pregnancy, AED, and HAART intersect. While it was assumed that dyslipidemia in pregnancy is physiological and non-atherogenic [68], there is a mounting body of evidence suggesting an association with increased risks of preterm delivery [69, 70], gestational diabetes [71], and preeclampsia [72, 73]. Pregnant WWE on EI-AEDs may be at higher risk of dyslipidemia with studies from HIC suggesting an association with carbamazepine use [74–78]. Similarly, HAART has been implicated in dyslipidemia [79–81] during pregnancy, and especially and PI intake [82].

Seventy-five articles exploring dyslipidemia in HIV+ individuals on HAART were retrieved, of which one meta-analysis [42] and a retrospective study [43] were eligible. A meta-analysis estimated the overall prevalence of total cholesterol concentrations (25.47%; 95% CI 19.41–32.03%) in the HIV+ population on HAART, low HDL cholesterol (45.6%; 95% CI 34.11–57.30%), and elevated LDL cholesterol (24.30%; 95% CI 14.71–35.38%) and triglycerides (19.68%; 95% CI 14.77–25.09) [42].

Only one study studied HAART use in pregnant women. A matched case control study of 154 pregnant and 151 non-pregnant on HAART in Nigeria reported a higher mean of very-low-density lipoprotein milligrams per deciliter in pregnant women 67.79 (SD 162.75) versus non-pregnant women (*p* = 0.045) [43].

### Congenital malformation

No study was identified reporting on CM among HIV+ WWE on HAART and AEDs in SSA.

CM, pregnancy, AED, and HAART intersect. Pregnant HIV+ WWE on AEDs may be at risk for CM. Different antiepileptic drugs and dosages have different teratogenic risks [30]. In contrast, a meta-analysis on the safety of perinatal HAART did not report an increased risk of CM [83], although further studies are warranted [84].

We retrieved 95 articles which examined CM related to HAART exposure in SSA, of which four prospective studies [44–47], one retrospective study [48], one RCT [49], and a meta-analysis [50] were eligible.

There was no meta-analysis on the burden of CM in HIV+ pregnant women on HAART. The evidence for a higher risk of CM associated with nevirapine appears inconclusive. A study in South Africa found that first trimester exposure to nevirapine was associated with an increased risk of CM (risk ratio 9.28; 95% CI 2.3–37.9) when compared to no exposure [44]. A HAART registry in South Africa reported a prevalence of major CM of 6.4% [45], within the ranges of the general population (95% CI 2.5–8%) [85].

In SSA, efavirenz forms the preferred first-line HAART [86]. A meta-analysis found no differences in overall risks of CM between women on efavirenz versus non-efavirenz-containing regimens during the first trimester of pregnancy (relative risk 0.78; 95% CI 0.56–1.08) [50]. In line with these findings is a retrospective study, where no visible CM was observed in women exposed to either efavirenz or nevirapine [48]. In South Africa, a study showed that CM rates in births exposed to efavirenz during the first trimester were similar to births not exposed to HAART [44]. Moreover, a RCT among Ugandan/Zimbabwean HIV+ women did not observe any CM with in utero tenofovir exposure [49].

Nevertheless, amidst the transitioning to dolutegravir-based first-line regimens, there have been concerns about its safety in pregnancy. A preliminary analysis in Botswana suggested a higher prevalence of neural-tube defects with dolutegravir treatment at conception than with non-dolutegravir HAART at conception (difference 0.20%; 95% CI 0.01–0.59%) or with other types of HAART exposure [47]. A final analysis covering 90% of all births in Botswana suggested dolutegravir exposure to be less than 1% [46].

### Chronic kidney disease

No study examining the renal function of pregnant HIV+ WWE on HAART and AED in SSA was identified.

CKD, pregnancy, AED, and HAART intersect. Due to a suboptimal capacity of renal elimination, the dosing of AED in patients with CKD deserves special attention [87], especially with levetiracetam, which is nearly 100% cleared by renal excretion [88]. Women with CKD are less able to make the renal adaptations needed for a healthy pregnancy [89], which is all the more problematic as a pregnancy is already associated with hormonal changes that directly and indirectly alter renal function [90]. CKD occurs both as a frequent complication of HIV infection and as a consequence of HAART and its complications [91], particularly in the patients on tenofovir [92], a commonly used in SSA [93], and which is a first-line regimen for all HIV+ pregnant and breastfeeding women initiating lifelong HAART independent of CD4+ count [94]. If not managed, HIV-associated kidney disease may progress to renal failure, which is a main predictive factor of complications in pregnant women [95].

Two hundred and five articles examined CKD in HIV+ individuals on HAART, of which one meta-analysis [51] and a systematic review [52] were eligible. The pooled prevalence of CKD among HIV+ individuals on HAART was estimated to be 6.42% (95% CI 5.2–7.6%) [51].

There was no study examining risk factors for CKD in pregnant women on HAART in SSA. No studies were retrieved on CKD in pregnant women on HAART, and the only evidence stems from a systematic review reporting on tenofovir-based regimens in a general population, concluding that its clinical significance was not enough to contraindicate its continued use [52].

### Neurological development

No study examining the risk of neurological disorders in children having been exposed in utero to a combination of HAART and AED in SSA was retrieved.

Neurological disorders, AEDs, HAART, and pregnancy intersect. As AEDs are able to cross the placenta, they may be associated with adverse neurodevelopment outcomes [31]. A meta-analysis of 29 observational cohorts carried out in HIC found valproate alone or in combination with another AED to be associated with the highest odds of adverse neurodevelopmental outcomes compared to women who did not receive AED during pregnancy (OR 7.40; 95% CI 3.00–18.46) [31]. Furthermore, among the first and second generation broad-spectrum AEDs targeting adults, lamotrigine and valproate (OR 8.88, 95% CI 1.28–112.00; OR 17.29, 95% CI 2.40–217.60, respectively) were associated with an increased occurrence of autism compared to the same controls [31]. As endemic undernutrition and co-infections may compound any potential adverse effect of maternal HAART exposure, its potential effect in SSA is unclear [96].

We retrieved 50 articles on neurological disorders related to HAART in SSA, of which five prospective studies [53–57] were eligible. No meta-analyses were retrieved reporting on the burden on neurological disorders following in utero exposure to HAART. Different endpoints and conflicting data were found regarding the impact of in utero exposure to maternal HAART. One cohort study found HIV-exposed/uninfected children exposed in utero to efavirenz-based HAART to be at higher risk for neurodevelopmental disorders than HIV-exposed/uninfected children exposed to non-efavirenz-based HAART [53]. Another cohort study suggested HIV-exposed/uninfected children exposed to HAART were at increased odds of receptive and expressive language delays at 2 years of age [56]. In contrast, a RCT study using different control groups (dual-NRTI + PI-based versus a triple-NRTI-based HAART regimen [57]) and two observational cohort studies (maternal HAART-exposed children versus HIV unexposed uninfected children [55] and HIV-exposed uninfected children exposed in utero to HAART versus zidovudine monotherapy [54]) did not exhibit significant differences in neurodevelopmental outcomes.

## Discussion

Our review underscores the absence of studies related to our PICO population and the paucity of studies related to HIV+ pregnant women. The pooled burden of all six clinical aspects was not estimated for the HIV+ pregnant population (on HAART or without) and, for the general HIV+ population on HAART, was absent for SE, SJS, CM, and neurological outcomes. Furthermore, there were no studies retrieved reporting on CKD in pregnant HIV+ HAART. This review highlights the need for a better understanding of clinical responses to first-line HAART in pregnant women in SSA and how the concomitant use of HAART and first-line/second-line AED may affect pregnancy, fetal, and neurological outcomes.

With high AED treatment gaps in SSA as well as suboptimal AED adherence, it is likely that HIV+ WWE exhibit significant fluctuations in therapeutic ranges, which may result in SE [58], hence rendering management of epilepsy in pregnant HIV+ women on HAART a clinical conundrum.

The maintenance of an optimal plasma concentration within the confinements of a therapeutic range is further complicated by the physiological changes experienced by WWE during pregnancy that may alter the pharmacokinetics of AEDs [97].

Levetiracetam, which has the best safety profile for HIV+ women [10], is unaffordable for many, and valproate, which is contraindicated during pregnancy, is used as a last resort when other AEDs have failed to control seizures [98]. Switching from branded AEDs to generic AEDs, which may alter the absorption parameters of the drug along with its plasma level, is discouraged due to mounting evidence suggesting an association with poor outcomes [99].

It may be posited that switching between different first-line and second-line AEDs may entail larger fluctuations in pharmacokinetics. However, if a HIV+ WWE enters her pregnancy treated with valproic acid, it may still be clinically judicious to switch to a less teratogenic second-line AED as soon as possible provided that a gradual buildup of the added drug before weaning down the dose of the initial drug is ensured, while considering the unique physiological changes of pregnant WWE.

As EI-AEDs and lamotrigine use have been shown to entail a higher risk of SJS/TEN, to ensure safe management of seizures in HIV+ women, it is pivotal to assess the accrued risk due to HAART uptake. With the phasing out of nevirapine from HAART protocols, one can expect a reduction in the incidence of SJS/TEN in pregnant HIV+ WWE. Studies are required to determine if the severity of SJS/TEN impacts maternal and/or fetal outcomes and develop a validated SJS/TEN severity-of-illness score for this population, which should also consider the drugs implicated in SJS/TEN and commonly used by HIV+ individuals on HAART, including antibiotics [100], cotrimoxazole [39], malarial drugs [100], TB drugs [37, 39], and sulphonamides [37, 38, 100].

In utero exposure to valproic acid carries an unacceptable risk of CM and along with lamotrigine appears to carry a strong risk of autism, greatly augmented when used in combination [31]. More homogenous studies with similar endpoints are required to corroborate the emerging evidence of a higher risk of CM and possibly neurological impairment that dolutegravir and first-line HAART drugs may entail. It is also important to consider that in SSA, a non-negligible proportion of seizures may be partial or focal in nature; most of the latter being attributable to central nervous infections [101], which can cause structural damage in the brain. Such seizures have a varying response to AED treatment and can even be drug resistant, particularly in individuals with temporal abscesses [102]. Consequently, increasing the AED dose or switching to polytherapy in attempt to control refractory focal seizures may entail a heightened risk of teratogenicity as congenital malformations have been shown to increase in an AED dose-dependent and synergistic manner [103, 104].

The burden of dyslipidemia in the HIV+ general population on HAART is high, and in pregnant women, HAART intake, especially PI-based regimens, along with EI-AED intake is likely to conspire to increase the risk for maternal dyslipidemia. In this regard, the benefits of statins, known to reduce progression of vascular disease, in HIV+ adults on HAART [105] should be weighed against findings showing no clear relationship of CM with its use but cautioning against use in pregnancy [106].

The burden of CKD in the HIV+ general population on HAART is non-negligible and likely to be higher in pregnant women, in whom renal function decline may be accelerated. In the absence of studies reporting on renal parameters in this population, findings on the safety of tenofovir can only be extrapolated from a general HIV population in SSA [52] and from studies on pregnant women from HIC suggesting that there were no significant effects of its exposure on renal function [107]. In SSA, proper management of seizures in pregnant HIV+ WWE requires knowledge of the epidemiology of HIV co-infections in the region and of potential interactions with AED. Treating pregnant HIV+ WWE entails the challenge of considering potential pharmacokinetic interactions between HAART, AEDs, anti-malarial and TB drugs, and cotrimoxazole when determining efficacious and safe dose adjustments, while also considering their changing kidney physiology.

A case for enhanced availability of 2nd-line AED can be made on public health grounds due to the potential of EI-AED to lead to HAART-resistant strains [108], which may be transmitted vertically also through breastfeeding [109]. In addition, their potential interactions with other anti-infective treatments may lead to drug resistance within the community. Thus, the ultimate public health goal in SSA should be to treat all HIV+ women of reproductive age with epilepsy with levetiracetam.

In light of the scarcity of specific evidence-based epilepsy management guidelines, prevalence studies and cohort studies following HIV+ WWE over time and across pregnancies are needed to capture the cumulative effect of treatments in all identified clinical aspects, including barriers for concomitant HAART and AED adherence. This requires training of healthcare professionals in the management of SJD/TEN, renal function, dyslipidemia, and CM. Furthermore, operational research could bring insights into how enlarged PMTCT programs would be capable of accommodating epilepsy management.

### Strengths and limitations

The wide breadth of this scoping review captures the evidence and knowledge gaps which had hitherto not yet been synthesized. However, we acknowledge a number of limitations. No papers were found concerning the pre-specified target population. In light of the preponderance of studies examining clinical outcomes in a general HIV population on HAART, along with a heterogeneity of study designs and controls, a cautionary approach should be employed when extrapolating findings to our study population to develop contextualized, evidence-based clinical guidelines.

## Conclusions

There is a marked absence of studies for our PICO study population despite the urgency for evidenced-based clinical management guidelines for pregnant HIV+ WWE in SSA. Insights into how interactions between first- and second-line AEDs and newer antiretroviral agents play out in determining pregnancy and fetus outcomes should be sought, in particular how it impacts CKD, dyslipidemia, and CM. Efforts should be targeted at developing a validated SJS/TEN severity-of-illness score and a better understanding of the barriers to AED adherence. In light of the HIV burden in SSA, and the public health impact of interactions between EI-AEDs and first-line HAART, clinical guidelines should advocate for second-line AED.

## Supplementary information

**Additional file 1.** Annex 1.

**Additional file 2.** Annex 2.

## Data Availability

Yes

## Abbreviations

AIDS: Acquired immune deficiency syndrome
aOR: Adjusted odds ratio
AED: Antiepileptic drug
CKD: Chronic kidney disease
CI: Confidence interval
CM: Congenital malformation
EI-AED: Enzyme-inducing antiepileptic drug
HAART: Highly active antiretroviral therapy
HIC: High-income countries
HDL: High-density lipoprotein
HIV: Human immunodeficiency virus
LDL: Low-density lipoprotein
LIC: Low-income countries
NRTI: Nucleoside reverse transcriptase inhibitor
PI: Protease inhibitors
RCT: Randomized controlled trial
SJD: Stevens-Johnson syndrome
TEN: Toxic epidermal necrolysis
TB: Tuberculosis
WHO: World Health Organization
WWE: Women with epilepsy
SSA: Sub-Saharan Africa
PMTCT: Prevention of mother-to-child transmission
