# Supplementary material for: Epilepsy management in pregnant HIV+ women in sub-Saharan Africa, clinical aspects to consider: a scoping review
Source: BMC Med. 2020 Nov 17;18:341. doi: 10.1186/s12916-020-01799-0 (PMC7670685; doi:10.1186/s12916-020-01799-0)
Supplement: Supplementary file 1 — Additional file 1. Annex 1. [file 12916_2020_1799_MOESM1_ESM.docx]

The following search terms were used for a specific search targeting pregnant HIV+WWE and a more general search:

**Status epilepticus**

Status epilepticus AND HIV AND Africa (n=6)

*Anti epileptic drugs adherence*:

Antiepileptic drugs adherence AND pregnan* HIV AND Africa (n=0)

Antiepileptic drugs adherence AND HIV AND Africa (n=2)

**SJS/TEN:**

Steven Johnson Syndrome OR Toxic Epidermal Necrolysis AND pregnan* AND antiretroviral therapy AND antiepileptic drugs AND Africa” (n=0)

Steven Johnson syndrome OR Toxic Epidermal Necrolysis AND HIV AND pregnan* AND Africa: (n=5)

Steven Johnson Syndrome OR Toxic Epidermal Necrolysis AND antiretroviral therapy AND Africa (n=26)

Steven Johnson Syndrome OR Toxic Epidermal Necrolysis AND Africa= (n=109)

**Dyslipidemia:**

Dyslipidemia AND pregnan* AND antiepileptic drugs AND antiretroviral AND Africa (n=0)

Dyslipidemia AND antiretroviral therapy AND pregnan* AND Africa: (n=2)

Dyslipidemia AND antiretroviral therapy AND Africa: (n=85)

Dyslipidemia AND HIV AND Africa: (n=114)

**Congenital malformation:**

Congenital malformation AND antiretroviral drugs AND anti epileptic drugs AND Africa: (n=0)

Congenital malformation AND antiretroviral drugs AND anti epileptic drugs AND Africa (n=0)

Congenital malformation AND antiretroviral drugs AND Africa: (n=9)

Congenital malformation AND HIV AND Africa: (n=95)

**Chronic Kidney disease:**

Chronic Kidney disease AND antiretroviral drugs AND pregnan* AND Africa AND

antiepileptic drugs: (n=0)

Chronic Kidney disease AND HIV AND pregnan* AND Africa: (n=5)

Chronic Kidney disease AND antiretroviral therapy AND Africa (n=84)

Chronic Kidney disease AND HIV AND Africa” (n=205)

**Neurological disorders:**

In utero exposure AND antiretroviral therapy AND antiepileptic drugs AND Africa: (n= 0)

in utero exposure AND antiretroviral therapy AND Africa (n=50)
